# Cavity-BOX SOI: Advanced Silicon Substrate with Pre-Patterned BOX for Monolithic MEMS Fabrication

**DOI:** 10.3390/mi12040414

**Published:** 2021-04-08

**Authors:** Marta Maria Kluba, Jian Li, Katja Parkkinen, Marcus Louwerse, Jaap Snijder, Ronald Dekker

**Affiliations:** 1 The Electronic Components, Technology and Materials (ECTM) Group, Delft University of Technology, Mekelweg 5, 2628 CD Delft, The Netherlands; M.M.Kluba@tudelft.nl; 2 Research and Development, Technology, Okmetic Oy, Piitie 2, FI-01510 Vantaa, Finland; Katja.Parkkinen@okmetic.com; 3 MEMS & Micro Devices, Philips, High Tech Campus 4, 5656 AE Eindhoven, The Netherlands; Marcus.Louwerse@philips.com (M.L.); Jaap.Snijder@philips.com (J.S.); 4 Philips Research, High Tech Campus 34, 5656 AE Eindhoven, The Netherlands

**Keywords:** SOI substrate, cavity-SOI, cavity-BOX, patterned BOX, buried hard-etch mask, flex to rigid (F2R), MEMS, miniaturization, DBS, foldable devices

## Abstract

Several Silicon on Insulator (SOI) wafer manufacturers are now offering products with customer-defined cavities etched in the handle wafer, which significantly simplifies the fabrication of MEMS devices such as pressure sensors. This paper presents a novel cavity buried oxide (BOX) SOI substrate (cavity-BOX) that contains a patterned BOX layer. The patterned BOX can form a buried microchannels network, or serve as a stop layer and a buried hard-etch mask, to accurately pattern the device layer while etching it from the backside of the wafer using the cleanroom microfabrication compatible tools and methods. The use of the cavity-BOX as a buried hard-etch mask is demonstrated by applying it for the fabrication of a deep brain stimulation (DBS) demonstrator. The demonstrator consists of a large flexible area and precisely defined 80 µm-thick silicon islands wrapped into a 1.4 mm diameter cylinder. With cavity-BOX, the process of thinning and separating the silicon islands was largely simplified and became more robust. This test case illustrates how cavity-BOX wafers can advance the fabrication of various MEMS devices, especially those with complex geometry and added functionality, by enabling more design freedom and easing the optimization of the fabrication process.

## 1. Introduction

**Figure 1:**
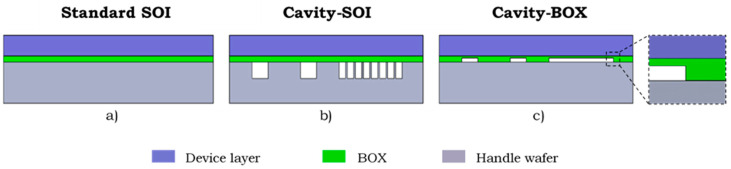
A comparison of three substrate architectures: (**a**) Standard SOI substrate; (**b**) Cavity-SOI substrate with cavities in handle wafer; (**c**) Cavity-BOX substrate with the pre-patterned buried oxide layer.

Standard SOI substrates were originally developed to enable perfect dielectric isolation in electronic devices. Nowadays, SOI wafers have also become an important substrate material for the fabrication of MEMS devices. SOI wafers consist of a handle wafer that provides mechanical strength during the fabrication process, a device layer in/on which the devices are fabricated, and a buried oxide (BOX) layer that separates the device layer from the handle wafer (see Figure 1a). Apart from electrical isolation, the BOX layer also allows for the fabrication of MEMS devices with a well-defined device layer thickness, and it can serve as a release layer in floating structures. SOI wafers are used in a wide range of applications, such as pressure sensors [1], resonators and inertial sensors [2], microchannels [3], and miniaturization of microfabricated medical devices [4,5].

Cavity-SOI substrate is a substrate that has been derived from the silicon-on-insulator-family [6]. It is a customized SOI substrate whereby the manufacturer of the SOI substrates has integrated customer-defined buried cavities in the silicon handle wafer (see Figure 1b). It has been demonstrated that customized SOI substrates with prefabricated cavities can significantly simplify the fabrication process of complicated MEMS devices, such as pressure or inertial sensors [2,7,8,9,10]. The cavity-SOI substrate allows for eliminating the cumbersome step of etching cavities in the handle layer later in the process flow, and permits for pre-patterning of complex cavities systems. However, the range of the cavity dimensions in the cavity-SOI wafers is limited. On the one hand, very large cavities that are not supported with pillars would make the wafer fragile and could lead to wafer or device layer deformation. On the other hand, very small buried structures are out of cavity-SOI scope, due to the low alignment precision of the prefabricated cavities with the structures fabricated later on the device layer.

Cavity-BOX is an advanced substrate with custom-defined cavities etched in the buried oxide (see Figure 1c). It is the newest member of the SOI substrate family. Its exemplar preparation process and application are presented in this paper. The cavities can be formed by etching through the complete thickness of the BOX or by partially etching the BOX to create a hard-etch mask with a step. In cavity-BOX substrates, only the thin layer of buried silicon oxide is patterned, which enables almost unlimited design of the cavities without weakening the mechanical properties of the wafer. The high-precision alignment (<500 nm) of the prefabricated cavities with the structures fabricated later, on the device layer, is ensured by applying a newly developed marker transferring strategy. The method uses a set of primary alignment markers, located on the SOI wafer terrace, that are transferred onto the device layer using front-to-front alignment [11]. The patterned BOX can serve as a stop layer during the device layer thinning, and it can be used as a hard mask during the device layer patterning from the backside. The high resolution of the deep reactive-ion etching (DRIE) process is maintained by bringing the hard mask, formed by the patterned BOX layer, directly to the device layer. This allows for a precise definition of micron-sized cavities in the device layer and, simultaneously, enables patterning centimeter-sized structures in the device layer. This paper presents the novelty of cavity-BOX substrates and illustrates how such a substrate can improve the fabrication of various MEMS devices. The use of the cavity-BOX as a stop layer and a buried hard-etch mask is demonstrated by applying it for the fabrication of a deep brain stimulation (DBS) demonstrator. First, the design and standard fabrication process of such a DBS device are presented and compared with the process that uses the cavity-BOX to show how the cavity-BOX substrate can enable more design freedom and simplify the fabrication process. Next, the preparation of the customized cavity-BOX substrate and the fabrication process of the DBS demonstrator are described. The DBS demonstrator is a mechanical structure composed of only silicon islands connected with a polymer-based flexible film. Finally, the DBS demonstrator fabrication results are presented and discussed.

## 2. Deep Brain Stimulation (DBS) Probe-Process and Design

**Figure 2:**
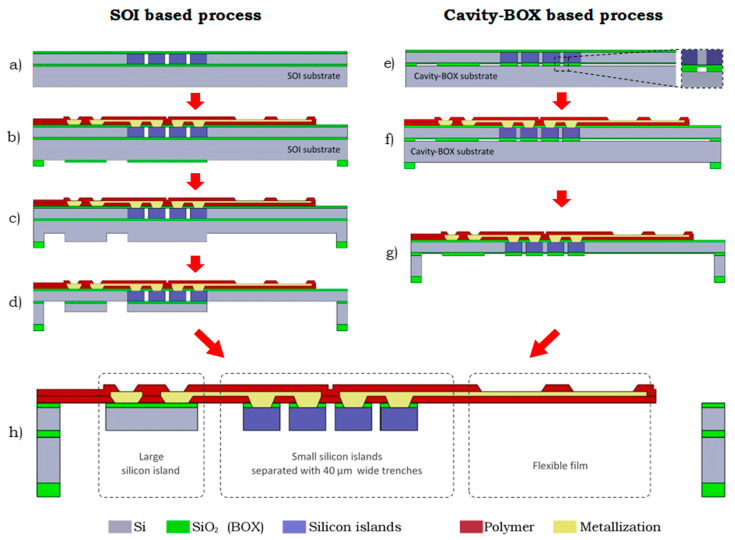
Simplified manufacturing process flow diagrams of semiflexible DBS device using the trench-based F2R technology versus using cavity-BOX substrate. Left (**a**–**d**): The SOI based process with sealed trenches on the front side of the wafer and a two-step backside etch process. Right (**e**–**g**): The cavity-BOX based process using patterned BOX as an etch-stop layer and hard-etch mask. Bottom (**h**): Finished device.

The advanced SOI substrate with cavities in the BOX can significantly simplify processes, such as microfabrication of highly integrated foldable devices [4,5] or 3-dimensional circuit integration using TSVs, in the device layer [12]. An example of the cavity-BOX application is a monolithic fabrication process of a foldable deep brain stimulation (DBS) device (see Figure 2 [5]).

Initially, standard SOI substrate and trench-based F2R technology were employed to accomplish the monolithic fabrication of a device where small 80 µm-thick silicon islands, separated with 40 µm-wide trenches, could coexist with a millimeter-sized flexible area etched from the backside of the wafer [5,13]. Due to the resolution limitations of the backside DRIE process, the precise separation of the small (210 × 2070 µm) silicon islands cannot be achieved. Therefore, the HAR trenches were etched in the device layer from the front side of the wafer to separate the silicon islands, and, subsequently, sealed with a silicon dioxide membrane (Figure 2a) to enable further wafer processing (Figure 2b). However, the trench etching and sealing processes require precise optimization, and they have very tight process windows. Moreover, failures of the fragile SiO_2_ membrane can severely hamper the follow-up processes. Employing the cavity-BOX substrate with patterned buried oxide (Figure 2e) allows for a more robust process by maintaining the device layer intact until the very end of the front side processing (Figure 2f). All the structures are later released by DRIE etching from the backside using the cavity-BOX as a hard mask.

After the front side processing is finished, the DRIE etching is applied from the backside of the wafer for thinning down and releasing the flexible structures. In the standard SOI process, this is realized by multiple steps of alternating silicon dioxide etch and silicon etch through a two-step hard-etch mask located on the backside of the handle wafer (Figure 2c,d,h). The buried oxide layer of the standard SOI wafer serves as an etch stop layer that defines the device thickness. This approach is cumbersome and heavily relies on the uniformity of each dry etching step. The cavity-BOX can significantly simplify the process by bringing it down to just three steps. First, the device is thinned down to 80 µm using a simple hard-etch mask patterned on the backside of the handle wafer, and the BOX as an etch stop layer that balances the DRIE uniformity of the handle wafer etching (Figure 2g). Secondly, the exposed cavity-BOX with a step mask is thinned down to form the hard-etch mask. Finally, the hard-mask formed in the BOX is used to separate the 80 µm-thick silicon islands with the 40 µm-wide trenches, and simultaneously release the flexible film in the final DRIE step (Figure 2h). The high resolution of the DRIE process and coexistence of the structures with a wide range of dimensions is ensured by bringing the hard mask–patterned BOX directly to the device layer, rather than optimizing the DRIE process to its extreme.

**Figure 3:**
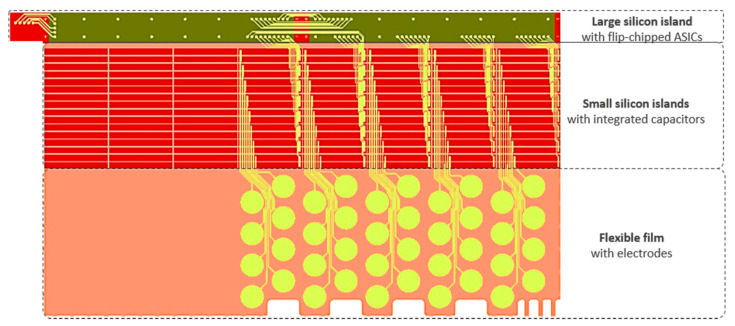
The 2D representation of the 40-electrode DBS design with integrated electronic components. The large silicon island (18 mm × 1 mm) can contain flip-chipped ASICs. The small silicon islands (210 µm × 2070 µm) are separated with 40 µm wide trenches and can accommodate prefabricated decoupling capacitors. The flexible film contains 40 flexible electrodes.

**Figure 4:**
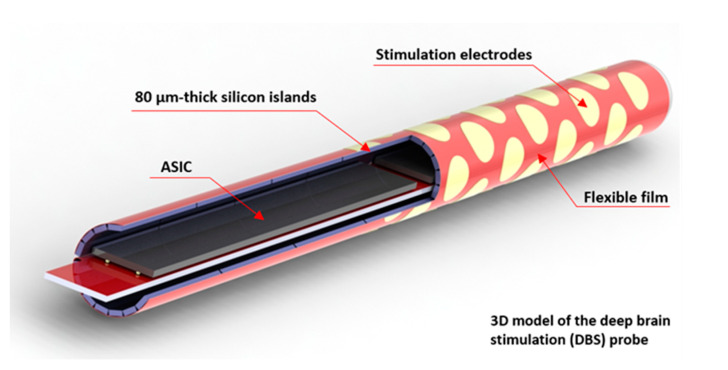
3D model of the 40-electrode DBS probe (ø 1.4 mm diameter) with integrated capacitors and ASICs inside the probe’s tip.

The application of the cavity-BOX substrate is demonstrated using a simplified fabrication process of an 18 mm long Deep Brain Stimulation (DBS) probe (see Figure 3). The highly integrated DBS tip was designed to accommodate 40 circular electrodes on a semi-film substrate. The small silicon islands can contain prefabricated decoupling capacitors, and the large silicon island permits for wire bonding and back-end integration of application specific integrated circuits (ASICs) inside the probe (e.g., flip-chip). All the structures are connected with flexible interconnects. This enables the activation of each electrode individually, using only a couple of power and signal wires reaching out of the probe. As a result of the semiflexible structure, consisting of multiple silicon islands connected with a flexible film, the device can be folded to a 1.4 mm diameter cylinder (Figure 4).

## 3. Fabrication

The DBS demonstrator was fabricated to illustrate the advances resulting from applying the cavity-BOX to the process. The DBS demonstrator presented here is a semiflexible mechanical structure, composed of silicon island and polymer-based flexible film, without interconnects or integrated electronic components.

The fabrication of the DBS demonstrator can be separated into two parts: the cavity-BOX SOI substrate preparation and the DBS demonstrator fabrication. The main technical challenge arises from the fact that the submicron (less than 1 µm) alignment accuracy between the buried cavity-BOX mask and the structures on top of the device layer must be guaranteed. To overcome that problem, the alignment marker transferring strategy, proposed and developed by C. Mountain et al. [11], was applied to ensure high precision alignment of the structures. Another goal was to demonstrate the functionality of the pre-patterned BOX mask.

### 3.1. Cavity-BOX Preparation

**Figure 5:**
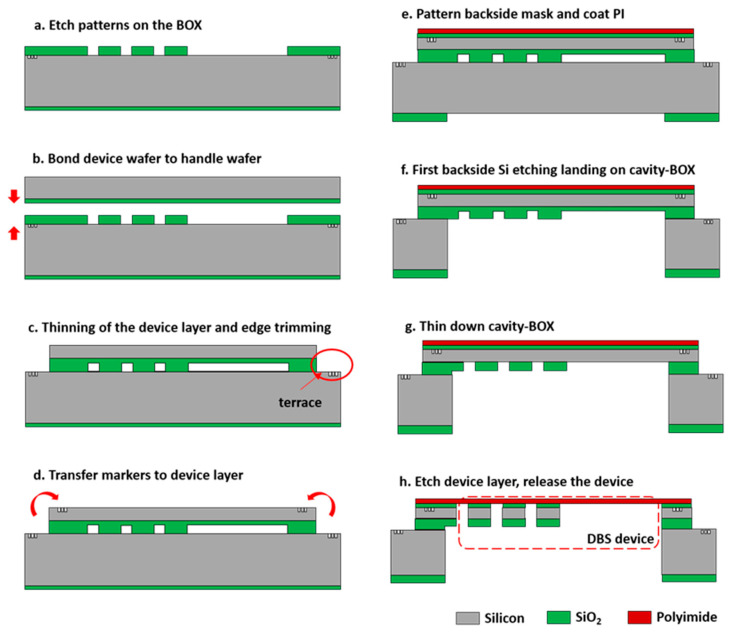
Cross-section drawings of the cavity-BOX substrate preparation and DBS demonstrator fabrication. (**a**) Positioning markers at 1.2 mm to wafer edge and etching patterns in the BOX on top of the 380 µm handle wafer. (**b**) Fusion bonding of the device wafer to the handle wafer with patterned BOX (in vacuum and room temperature. (**c**) Thinning of device layer to 80 µm and edge trimming to create a 4 mm width terrace. (**d**) Transfer markers from the terrace to the device layer. (**e**) Patterning backside etching mask and coating wafer with polyimide, using silicon oxide as an adhesive layer. (**f**) Etching handle wafer from wafer backside and landing on the cavity-BOX. (**g**) Thinning down the cavity-BOX and exposing the buried oxide mask for device layer etching. (**h**) Etching device layer and silicon oxide adhesion layer, landing on polyimide.

A schematic process flow of cavity-BOX substrate preparation is presented in Figure 5. A 380 µm-thick 6-inch double side polished (DSP) handle wafer was used as a starting material. First, two 140 nm deep ASML markers were patterned into the silicon substrate, 1.2 mm away from the left and right edge of the wafer. Next, 1 µm of high-quality thermal SiO_2_ layer for wafer bonding was grown on both sides of the wafer. The customized cavity-BOX pattern was aligned with the markers, and dry etched into the SiO_2_ layer, landing on the silicon (Figure 5a). The handle wafer was subsequently fusion bonded with the device layer, which also had a 500 nm-thick layer of thermal oxide. The fusion bonding was carried out under vacuum at room temperature. The two oxide layers were bonded and merged into the cavity-BOX with the pre-patterned step oxide mask (Figure 5b). Finally, the device layer was thinned down to 80 µm, and a terrace with a width of 4 mm was created by a combination of edge trimming and wet etching (Figure 5c). The alignment markers on the handle wafer were revealed during that process.

The 4 mm terrace width was chosen to keep the edge of the device layer as far as possible from the alignment markers on the handle wafer, in case of any possible optical interference during the marker transferring processes. The cavity-BOX substrate is ready after the terracing process. It contained a patterned step buried oxide layer (1.5 µm at its full thickness, 500 nm at its step thickness), an 80 µm-thick device layer, and 1 µm thermal oxide on the backside of the wafer.

### 3.2. DBS Demonstrator Fabrication

To continue with the demonstrator fabrication, the handle wafer markers were first transferred to the device layer, placed 10 mm from the wafer edge, and etched 140 nm deep into the silicon (Figure 5d). A 2 µm-thick PECVD SiO_2_ layer was then deposited on top of the original 1 µm-thick thermal oxide layer on the backside of the wafer and patterned into the silicon DRIE etching mask. Next, a 500 nm-thick PECVD SiO_2_ layer was deposited on the front side of the wafer as an adhesion layer. Subsequently, a 3 µm-thick polyimide layer (PI2610 Microsystems) was coated on top of the SiO_2_ adhesion layer (Figure 5e) and cured. After that, the silicon DRIE etching step was applied from the backside of the wafer to remove the silicon substrate underneath the cavity-BOX landing on the step oxide mask (Figure 5f). The step oxide mask enabled the silicon over-etch to balance the etching nonuniformity across the wafer. An overall SiO_2_ dry etching was subsequently applied to thin down the step oxide mask in the cavity-BOX layer until the pre-patterned oxide mask was opened through to the device layer (Figure 5g). Finally, the 80 µm device layer and the 500 nm SiO_2_ layer used for polyimide adhesion were dry-etched, landing on the polyimide layer (Figure 5h). After the etching of the device layer, all the silicon islands were separated, and they were connected with the flexible polyimide film. The finished demonstrator was suspended in a silicon wafer frame through polyimide tabs.

## 4. Results and Discussion

Both the substrate preparation and the demonstrator fabrication were straightforward. The alignment markers on the terrace edge were successfully detected and transferred to the device layer (Figure 5d). However, there are several other critical steps in the process. The bonding result of the cavity-BOX while preparing for the substrate directly affects its functionality. The terrace width caused an unexpected compatibility problem. Moreover, the backside DRIE etching defines the final device. Here we discuss the aforementioned issues and present the fabricated and assembled DBS demonstrator.

### 4.1. Preparation of the Cavity-BOX

The cavity-BOX is formed by the bonding process of two oxide layers: 1 µm of pre-patterned SiO_2_ layer from the handle wafer and a 500 nm-thick SiO_2_ layer from the device layer, with the accuracy of 300 to 500 nm (Figure 5b). The potential concerns of the bonding process include the following: (1) Bonding failures of the small oxide features from the handle wafer; (2) undesired bonding between the silicon substrate of the handle wafer and the SiO_2_ layer from the device layer in the large dimension cavities; and (3) sunken surface of the device layer due to the cavities in the BOX.

**Figure 6:**
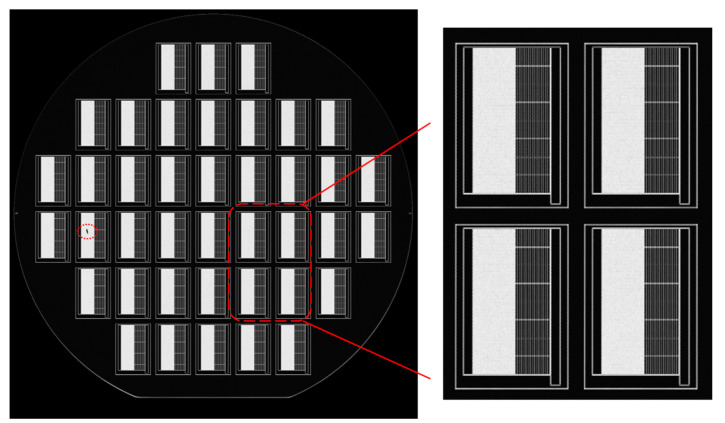
Scanning acoustic microscopy (SAM) to inspect bonding quality. A particle appeared as a black dot on the left side, and a zoomed-in SAM image of four dies.

After the cavity-BOX substrate preparation, no visible sunken surfaces were observed in the device layer. A scanning acoustics microscopy (SAM) was applied to inspect the bonding quality, as shown in Figure 6. The bonded area has a high transmission to the acoustic waves, which are displayed as dark fields in the picture, while air gaps have high reflections on the acoustic waves and are, therefore, displayed as bright fields. The images indicate that the area without cavities was successfully bonded, while there was no undesired bonding in the large cavity areas. No bonding defects have been observed among the small oxide features in the SAM images. Later, after the backside etching of the handle wafer landing on the cavity-BOX (Figure 5f), the two-step oxide mask was intact, which also confirms the excellent bonding result.

### 4.2. Terrace Width

During the backside processing, it appeared that the 4 mm terrace width on the front side of the SOI wafer caused compatibility issues with the PAS5500 ASML wafer stepper and the SPTS Pegasus DRIE etching tool. During the backside lithography process, the wafer stepper needed to handle the wafer from the front side. The positionings of a few vacuum pads on the wafer stepper robot arm were in the 4 mm terrace region, which led to a loss of vacuum. The wide terrace additionally caused helium leakage errors on the chuck of the dry etching tools. To continue processing the 4 mm terrace cavity-BOX SOI wafers, the robot arm of the wafer stepper was explicitly tuned to accept the wafers, and special dry etching recipes with low helium flow were developed for the silicon and SiO_2_ etch. As a result of reducing the helium flow in the etching recipes, the wafer temperature could not be well maintained during the process, hence, leading to an increased nonuniform etch rate across the wafer. The etching slop was also enlarged due to the loss of temperature control.

Therefore, the terrace width is preferred to be as small as possible for cleanroom compatibility. In contrast, the terrace edge should be as far as possible from the markers to avoid its interference with the alignment process, requesting a large terrace width. A trade-off test was performed to define the optimal terrace width. Wafers with different terrace widths were tested in the standard 6-inch cleanroom processing line in the PAS5500 ASML wafer stepper, an SPTS Pegasus, ICP, and APS etching tools. The test results indicate that the terrace width of 1.8 to 2.5 mm should provide sufficient compatibility.

### 4.3. Backside Etching of the Demonstrator

To release the demonstrator and form the semiflexible device structure, several etching steps were performed from the backside of the wafer. These etching steps include the following: (1) The DRIE etching of the handle wafer substrate landing on the cavity-BOX; (2) thinning down the cavity-BOX to form the pre-patterned oxide mask; (3) etching of the device layer and the polyimide adhesion oxide layer using the oxide mask (Figure 5f,h).

**Figure 7:**
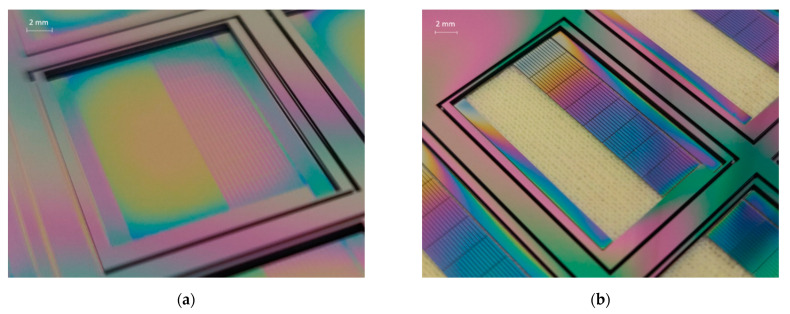
(**a**) Backside etching result of a silicon substrate, landing on cavity-BOX. (**b**) Etching device layer while using the patterned cavity-BOX as a mask, landing on polyimide.

Figure 7a depicts the wafer after the first step of the backside Si etching process landing on cavity-BOX. The device layer was protected by the exposed step cavity-BOX. The thickness of the remaining step cavity-BOX after silicon etching was measured using a reflectometer (Nanospec). The thinner part of the step cavity-BOX ranged from 120 nm to 350 nm (originally 500 nm), and the thicker part ranged from 1150 nm to 1380 nm (originally 1500 nm). It can be concluded that the first silicon etching successfully landed on the cavity-BOX, which functions as an etch-stop layer. Figure 7b presents the wafer with the etched device layer after the third backside etching step. The large rectangular opening was transparent, as the etching landed on the polyimide layer coated on the front side of the wafer. All the silicon structures, including the 40 µm-wide trenches between the silicon islands and the gaps that defined the silicon frame, were well fabricated.

**Figure 8:**
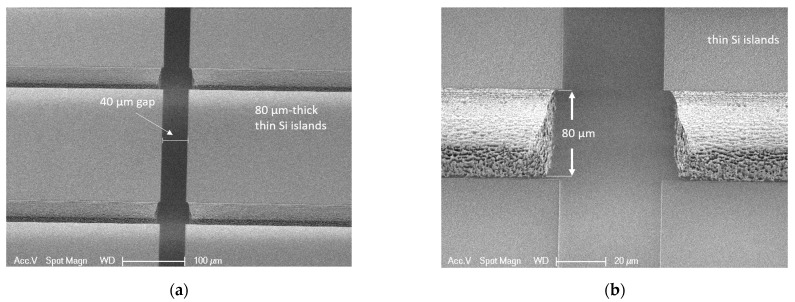
(**a**) SEM image of the 40 µm gaps between the silicon islands. (**b**) Zoom-in image of the 80 µm-thick device layer etching profile, with 6,3 µm under-etch from each side.

Figure 8 shows the SEM images of the silicon islands separated with the 40 µm trenches. The top part of the trench between the small silicon islands is 41.4 µm wide, while the bottom part is 54 µm wide. The measurement indicates an under etch of 6.3 µm from each side during the device layer etching, which is not ideal considering the fact that the etching depth is only 80 µm. The large under etching was mainly caused by temperature control issues, which resulted from the lowered helium flow that was necessary to process the wafer on the chuck of the silicon etcher. A better etching profile can be achieved for wafers with compatible terrace width that can be etched using the standard DRIE process without modifying the cooling gas flow.

### 4.4. Demonstrator Assembly

**Figure 9:**
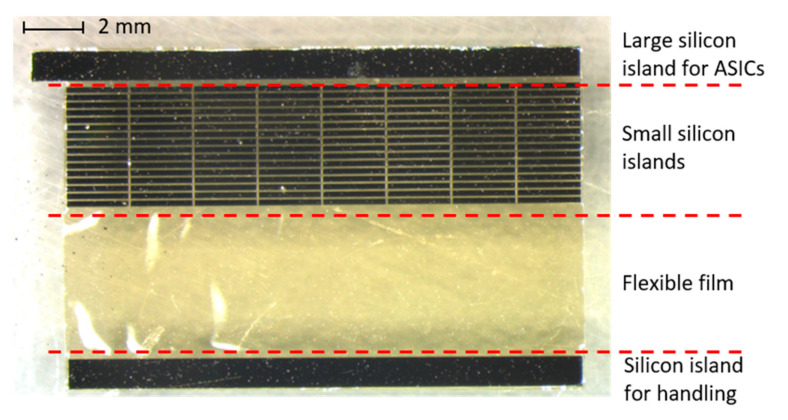
DBS demonstrator released after the fabrication. All the 80 µm-thick silicon islands are connected by the flexible film. The small silicon islands are isolated by 40 um-wide gaps.

**Figure 10:**
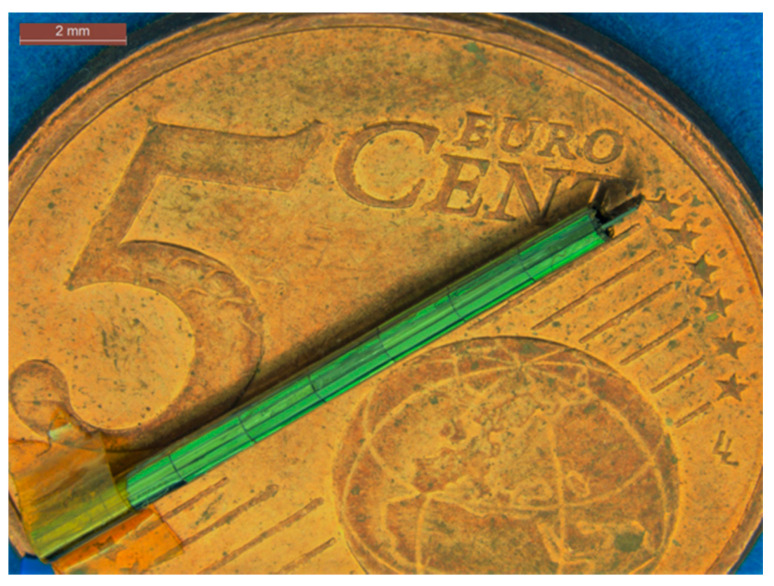
DBS demonstrator wrapped into a cylindrical probe with a length of 18 mm and a diameter of 1.2 µm.

After fabrication, the demonstrator was taken out of the silicon frame (see Figure 9). The demonstrator consists of one large silicon island that can accommodate ASICs, 128 small silicon islands with 40 µm gaps between them, a large flexible film, and a silicon island for handling. In the final DBS device, the decoupling capacitors are located on each small silicon island, and the electrodes are located on the flexible polyimide film. During the assembly process, the silicon island for the ASICs was attached to a thin metal string with a double-side adhesive Kapton tape. The metal string was slowly rotated, together with the silicon piece, tightening up and wrapping the semiflexible device into a cylindrical probe. In Figure 10, the assembled demonstrator has a length of 18 mm and a diameter of 1.2 mm, which is in line with the DBS design.

## 5. Conclusions

Advanced cavity-BOX SOI substrates with a buried oxide mask were developed, prepared, and applied in the process of a semiflexible microfabricated device. The oxide mask was first fabricated on the handle wafer and then bonded with the oxide on the device layer to form the cavity-BOX. The alignment between the structures on the device layer and the buried oxide mask was successfully realized by employing the front-to-front markers transferring strategy. The advanced SOI substrate was implemented for a DBS demonstrator fabrication. The cavity-BOX layer was used as an etch-stop layer and a pre-patterned two-step DRIE mask to etch through the device layer from the backside of the wafer. This two-step oxide layer was successfully used to compensate for etch rate differences during the bulk of the silicon removal. The application of the cavity-BOX substrate to the DBS demonstrator fabrication proved a more robust and significantly simplified process and large design freedom, including the coexistence of high-precision micron-sized features and a large millimeter-sized opening. The semiflexible DBS demonstrator with a length of 18 mm and a diameter of 1.39 mm was successfully fabricated and assembled.

The cavity-BOX SOI has a great potential to simplify the fabrication of various MEMS devices, where device thinning and precise silicon structure separation/definition is needed. The cavity-BOX SOI substrate with a terrace width from 1.8 mm to 2.5 mm is compatible with standard cleanroom equipment for both the front and backside processes. This terrace width also permits applying the high precision (less than 500 nm) alignment strategy of the BOX pattern and the device layer structures. Depending on design requests, the customized cavity-BOX SOI can be provided by the commercial SOI suppliers in large volume, hence, extensively enabling the scaling up of the production.
